# OPN promotes pro-inflammatory cytokine expression via ERK/JNK pathway and M1 macrophage polarization in Rosacea

**DOI:** 10.3389/fimmu.2023.1285951

**Published:** 2024-01-05

**Authors:** Siyi Tang, Hao Hu, Manhui Li, Kaoyuan Zhang, Qi Wu, Xiaojuan Liu, Lin Wu, Bo Yu, Xiaofan Chen

**Affiliations:** ^1^ Shenzhen Key Laboratory for Translational Medicine of Dermatology, Biomedical Research Institute, Shenzhen Peking University - The Hong Kong University of Science and Technology Medical Center, Shenzhen, Guangdong, China; ^2^ Department of Dermatology, Peking University Shenzhen Hospital, Shenzhen, China; ^3^ Greater Bay Biomedical Innocenter, Shenzhen Bay Laboratory, Shenzhen, China

**Keywords:** Rosacea, OPN, keratinocyte, macrophage, inflammation

## Abstract

Rosacea is a chronic inflammatory dermatosis that involves dysregulation of innate and adaptive immune systems. Osteopontin (OPN) is a phosphorylated glycoprotein produced by a broad range of immune cells such as macrophages, keratinocytes, and T cells. However, the role of OPN in rosacea remains to be elucidated. In this study, it was found that OPN expression was significantly upregulated in rosacea patients and LL37-induced rosacea-like skin inflammation. Transcriptome sequencing results indicated that OPN regulated pro-inflammatory cytokines and promoted macrophage polarization towards M1 phenotype in rosacea-like skin inflammation. *In vitro*, it was demonstrated that intracellular OPN (iOPN) promoted LL37-induced IL1B production through ERK1/2 and JNK pathways in keratinocytes. Moreover, secreted OPN (sOPN) played an important role in keratinocyte-macrophage crosstalk. In conclusion, sOPN and iOPN were identified as key regulators of the innate immune system and played different roles in the pathogenesis of rosacea.

## 1 Introduction

Rosacea is a chronic inflammatory skin disorder primarily affecting the midface (1), which is characterized by recurrent flushing or persistent erythema, pustules, papules, phymatous changes, and telangiectasia (2). There are three clinical types of rosacea, including erythematotelangiectatic (ETR) type, papulopustular (PPR) type, and phytates (PhR) type (3). Recent research into the pathogenesis of rosacea suggested that dysregulation of the immune system as well as neurovascular played a key role in the development of rosacea (1). As the “initiator” of rosacea, activated keratinocytes secreted large numbers of inflammatory factors that function as important signal transmitters between keratinocytes and other immune cells. Multiple innate immune cells including macrophages, neutrophils, and mast cells (4), were infiltrated in rosacea lesions and contributed significantly to the pathophysiology of rosacea (5, 6). Moreover, proinflammatory cytokines are also key effectors of the inflammatory response in rosacea. Cathelicidin LL37 may induce the production of characteristic cytokines and chemokines via mTORC1 signaling pathway in rosacea (7). Due to its role in inflammation and angiogenesis, IL-1β is one of the most important pro-inflammatory cytokines in the pathogenesis of rosacea (8). However, there was need to demonstrate the mechanism of elevated IL-1β in rosacea.

As part of the innate immune system, macrophages differentiated from monocytes were recruited from the blood into inflamed tissues (9, 10), which play a vital role in regulating local inflammatory response. Macrophages are capable of changing their phenotypes in response to many different stimuli, including classically activated macrophages (M1 macrophages) and alternatively activated macrophages (M2 macrophages) (11). M1 macrophages are pro-inflammatory and play a central role in host defense against infection, whereas M2 macrophages are associated with anti-inflammatory responses and tissue remodeling (11). Macrophages regulate the initiation and progression of inflammatory diseases through the transformation of different phenotypes (12). Selective depletion of M1 macrophages in transgenic mice inhibits chronic skin inflammation (13), while selective activation of M2 macrophages ultimately attenuates atopic dermatitis (14). These studies illustrate that the reduction of the M1/M2 ratio suppresses skin inflammation. Recruitment and activation of macrophages have been reported in the skin lesions of rosacea patients (5), and the polarization of macrophages towards the M1 phenotype may exacerbate rosacea inflammation (15). Crosstalk between macrophages and keratinocytes may participate in macrophage polarization in rosacea pathogenesis. However, the exact signal transmitter between them still needs to be investigated in depth.

Osteopontin (OPN) is a phosphorylated glycoprotein produced by a broad range of cells including osteoclasts (16), T cells (17), macrophages (18), and fibroblasts (19). Previous studies have revealed the roles of OPN in the process of immune response (20), tumorigenesis (21), and biomineralization (22). Many studies have shown that secreted OPN (sOPN) has a chemotactic effect on macrophages and neutrophils during acute and chronic inflammation processes (23). However, another intracellular form of OPN (iOPN) without a signal sequence has recently attracted considerable attention in immune cell signaling (23). Different forms of OPN may exist in different cells (24). Which forms of OPN exist in keratinocytes and how they regulate the inflammatory signaling pathways and macrophage polarization in rosacea remain unclear.

In the present study, we focused on the role of epidermal OPN in the pathogenesis of rosacea. Firstly, it was found that OPN was upregulated in the epidermis of rosacea patients and promoted LL37-induced skin inflammation. OPN promoted rosacea-like skin inflammation by increasing the infiltration of macrophages and angiogenesis. Furthermore, it was demonstrated that iOPN promoted pro-inflammatory cytokine IL1B expression via ERK1/2 and JNK signaling pathways in keratinocytes, while sOPN played a key role in keratinocyte-macrophage crosstalk by promoting M1 macrophage polarization. These results indicated that OPN might be a promising therapeutic target in rosacea treatment.

## 2 Materials and methods

### 2.1 Datasets

The epidermal rosacea transcriptome dataset (GSE65914) of rosacea lesions and healthy controls was downloaded from the Gene Expression Omnibus (GEO) database (https://www.ncbi.nlm.nih.gov/gds/).

### 2.2 Patient and specimen collection

All skin biopsy samples (three rosacea patients and three healthy controls) included in this study were obtained from the Department of Dermatology, Peking University Shenzhen Hospital (Shenzhen, China). In this study, all the patients were not treated with systemic treatment before skin biopsy. The control groups were obtained from biopsies of non-inflamed skin from healthy subjects. The collected samples were fixed with 4% paraformaldehyde and embedded for tissue sectioning and staining. This study was conducted in accordance with the Declaration of Helsinki and approved by the Ethics Committee of Peking University Shenzhen Hospital. Informed consent was obtained from all participants.

### 2.3 Cell culture and treatment

HaCaT and THP-1 cells were obtained from the China Center for Type Culture Collection (China). HaCaT and THP-1 cells were cultured in DMEM (Gibco, USA) and RPMI-1640 (Gibco, USA) growth medium containing 10% FBS (Gibco, USA) in a humidified atmosphere with 5% CO_2_ at 37°C. For LL37 treatment, HaCaT cells were stimulated with LL37 (QYAOBIO, China) (80, 100μg/ml) in serum-free medium for 12 hours. For siRNA treatment, HaCaT cells at 70% confluence were transfected with small interfering RNA targeting OPN mRNA (si-OPN) or negative controls (si-NC) by Lipofectamine™ RNAiMAX Transfection Reagent (Thermo Scientific, USA) according to the manufacturer’s instructions. siRNAs were bought from Ribobio (China). The siRNA sequence that targeted OPN mRNA was as follows: 5’-GAACGACTCTGATGATGTA-3’. For inhibitor treatment, ERK1/2 inhibitor (SCH772984) (MedChemExpress, USA) and JNK inhibitor II (Thermo Fisher Scientific, USA) were used to inhibit ERK1/2 and JNK signaling pathways in HaCaT cells. THP-1 cells were incubated with 50 ng/ml phorbol 12-myristate 13-acetate (PMA; Sigma-Aldrich, USA) for 48 hours to differentiate into macrophages (M0), and then stimulated with 1000ng/ml recombinant human OPN (rhOPN) (Sino Biological, China) for 48 hours. The endotoxin level of rhOPN is less than 1.0 EU/μg as determined by the LAL method in the manual from its manufacturer.

### 2.4 Animal experiments

OPN knockout (OPN KO) mice on C57BL/6 background were obtained from The Jackson Laboratory (USA). Female C57BL/6 wild-type (WT) mice (6-8 weeks of age) were kept under specific pathogen-free conditions at 24°C. All procedures were approved and supervised by Shenzhen Perking University - the Hong Kong University of Science and Technology Medical Center Animal Care and Use Committee. LL37 was synthesized in QYAOBIO (China). For the rosacea-like mouse model (23), the backs of the mice were shaved 24 hours before treatment, and then injected with LL37 by intradermal injection every 12 hours for 2 days to induce a rosacea-like inflammatory phenotype. Erythema on the lesions was photographed and evaluated 24 hours after the last injection.

### 2.5 RNA sequencing and analysis

RNA qualification, library preparation, sequencing, quality control, read mapping to the reference genome, and expression analysis were performed by BGI Genomics (Wuhan, China). In brief, the total RNAs from the mouse skin tissues were extracted with TRIzol reagent (Sigma-Aldrich, USA) according to the manufacturer’s instructions. The RNA samples were denatured at appropriate temperature to reveal its secondary structure, and oligo(dT) magnetic beads were used to enrich the mRNA. Fragmentation reagents were added to the mRNA to fragment the mRNA. Then one-strand and two-strand cDNA were synthesized. The ends of the double-stranded cDNA were repaired. A single “A” nucleotide is added to the 3’ end to connect the adapter to the cDNA, then the product is amplified. After the PCR product was denatured into a single-stranded product, a cyclization reaction system was prepared to obtain a single-stranded circular product and digest the uncirculated linear DNA molecules. Single-stranded circular DNA molecules replicated through rolling circles form DNA nanoball (DNB) containing multiple copies. DNBs were added to the mesh holes on the chip using high-density DNA nanochip technology, and sequenced through combined probe-anchored polymerization technology. To obtain clean data, the raw data were filtered with SOAPnuke. HISAT2 was used to align the clean data to the reference genome. Bowtie2 was used to align the clean data to the reference gene set. The expression levels of genes were calculated by RSEM.

Using |log2FC| ≥ 1, p value < 0.05 as the thresholds, differentially expressed genes (DEGs) were identified with the DESeq R package. Kyoto Encyclopedia of Genes and Genomes (KEGG) pathway analysis was performed by R clusterProfiler package. For immune infiltration analysis, relative levels of different immune cell types in WT mice and OPN KO mice were quantified by using the CIBERSORT algorithm (https://cibersort.stanford.edu).

### 2.6 Isolation and culture of primary mouse keratinocytes

C57BL/6 wildtype (WT) mice and OPN knockout (OPN KO) mice neonates from post-natal days 0 to 2 were sacrificed, of which limbs were removed then. After washed with 70% ethanol and PBS, the whole skins were peeled off and floated in ice-cold dispase digestion buffer overnight at 4°C. The next day, the epidermis and dermis were mechanically separated with forceps, and then CnT-Accutase-100 (CellnTec, Switzerland) was added to detach keratinocytes from the separated epidermis. The primary keratinocytes were seeded in the six-well plate with CnT-07 Epithelial Proliferation Medium (CellnTec, Switzerland) and CnT-07.S (CellnTec, Switzerland), which were cultivated at 37°C with 5% CO_2_ for 48 hours. All animal studies were approved and supervised by Shenzhen Perking University - the Hong Kong University of Science and Technology Medical Center Animal Care and Use Committee.

### 2.7 Generation of stable cell lines expressing sOPN and iOPN

The 903 nucleotide (nt) coding sequence (CDS) of sOPN was cloned into the lentiviral vector pLVX-IRES-Hyg (Addgene, USA), which encodes a polypeptide of 300 amino acids. Similarly, the 855 nt CDS of iOPN, lacking the first 48 nt signal peptide of sOPN, was cloned into the pLVX-IRES-Hyg vector. The nucleotide and protein sequences of OPN are listed in **Figure S1**, and primers used for cloning are shown in **Table S1**. An empty vector without fragment insert was also used to produce a control lentivirus. To generate sOPN and iOPN expressing lentivirus, the lentiviral vector Lenti-iOPN (LV-iOPN), Lenti-sOPN (LV-sOPN), or LV-negative control (LV-NC) co-transfected with psPAX2 and pMD2.G plasmids (Addgene, USA) into 293T packaging cells. 12 hours after transfection, cells were supplied with fresh medium and cultured for an additional 36 hours. The lentivirus-containing supernatant was filtered and used for cell infection. Then HaCaT cells were infected by the lentivirus mentioned above. After antibiotic selection for a week, the stable HaCaT cells expressing sOPN (LV-sOPN HaCaTs), iOPN (LV-iOPN HaCaTs), and LV-NC HaCaTs as negative control were obtained.

### 2.8 Co-culture assay

Firstly, the HaCaT cells and the THP-1 cells were cultured separately. THP-1 cells cultured in RPMI 1640 growth medium were seeded into transwell inserts (Corning, USA) with a 0.4μm pore size polycarbonate permeable membrane and differentiated with 50 ng/ml PMA for 48 hours. The LV-sOPN HaCaTs, LV-iOPN HaCaTs, and si-OPN-treated HaCaTs cultured in DMEM growth medium were seeded in 6-well plates and stimulated by LL37 (100μg/ml) for 24 hours in serum-free media. After washed with PBS, THP-1-derived macrophages were co-cultured with HaCaT cells for another 48 hours. Then macrophage mRNA was collected.

### 2.9 RNA extraction and real-time quantitative PCR

The total RNA was extracted with TRIzol reagent (Sigma-Aldrich, USA) according to the manufacturer’s instructions. RNA concentration was detected using Nanodrop-2000 (Thermo Scientific, USA). Reverse transcription was carried out with the High Capacity cDNA Reverse Transcription Kit (BioRad, USA). Then mRNA expression was determined with real-time quantitative PCR (qRT-PCR). The qRT-PCR reaction mix contained 5 μl SYBR Green qPCR Master Mix, 3 μl diluted primer mix, and 2 μl diluted cDNA. Bio-Rad CFX96 real-time fast PCR system was used to perform qRT-PCR amplification. β-Actin was an internal control, and the relative expression of mRNA was calculated according to the 2^-△△Ct^ method. All the sequences of primers used for qRT-PCR in this study are listed in **Table S1**.

### 2.10 Western blot analysis

Briefly, cells were harvested on ice with ice-cold RIPA lysis buffer containing protease inhibitor cocktails. Proteins were separated by 10% SDS-PAGE gel and transferred into PVDF membranes for western blot assay. The membranes were blocked with 5% BSA and then incubated with appropriate primary and horseradish peroxidase (HRP)-conjugated secondary antibodies. Finally, the immunosignals were detected using the SuperSignal West Femto Chemiluminescent Substrate Kit (Thermo Scientific, USA). Antibodies from Cell Signaling Technology (USA) are as follows: β-Actin (8H10D10) Mouse mAb (#3700), p44/42 MAPK (Erk1/2) (137F5) Rabbit mAb (#4695), Phospho-p44/42 MAPK (Erk1/2) (Thr202/Tyr204) Antibody (#9101), Phospho-SAPK/JNK (Thr183/Tyr185) (98F2) Rabbit mAb (#4671), SAPK/JNK Antibody (#9252), HRP-linked Antibody (#7074).

### 2.11 Flow cytometry

The skin samples of the LL37-induced rosacea mice model were cut into small pieces and processed in digestion buffer (1 mg/ml collagenase) for 2 hours at 37°C. Cells from the skin samples were passed through 40 μm nylon mesh to obtain a single-cell suspension, then the single cells were resuspended in 30% Percoll solutions and centrifuged. Subsequently, the single-cell suspension was stained with Zombie Aqua (Biolegend, USA) for 20 minutes, blocked with Fc block for 10 minutes (anti-mouse CD16/32 mAbs; Biolegend, USA), and finally stained with the fluorochrome-conjugated antibodies for 20 minutes. Cells were then washed and measured on CytoFLEX (Beckman Coulter, USA) flow cytometer and analyzed by CytExpert (Beckman Coulter, USA) software. M1 macrophages were defined as CD45^+^CD11b^+^F4/80^+^CD86^+^cells. M2 macrophages were defined as CD45^+^CD11b^+^F4/80^+^CD206^+^ cells.

The following antibodies from BioLegend (CA, USA) were used: FITC anti-mouse/human CD11b (M1/70), APC/Cyanine7 anti-mouse CD45 (S18009F), APC anti-mouse F4/80 (BM8), PE/Cyanine7 anti-mouse CD206 (C068C2), Brilliant Violet 421 anti-mouse CD86 (GL-1).

### 2.12 Histology and immunohistochemistry

Skin tissues were fixed in 4% paraformaldehyde, embedded in paraffin, and sectioned. Sections were stained with hematoxylin and eosin (H&E). For immunohistochemistry, skin tissues were dewaxed with xylene and hydrated with ethanol, then subjected to antigen retrieval and blocked endogenous peroxidase activity with 3% hydrogen peroxide. After incubation with blocking buffer for 1 hour at room temperature, tissue sections were incubated with primary antibody and peroxidase-conjugated secondary antibody (Beyotime Biotechnology, China). The staining results were visualized using SignalStain^®^ DAB Substrate Kit (Cell Signaling Technology Inc., USA) and finally counterstained with Mayer’s hematoxylin.

The following primary antibodies were used: Anti-Osteopontin antibody (ab8448) was from Abcam (USA). F4/80 (D2S9R) Rabbit mAb, CD4 (D2E6M) Rabbit mAb, and CD31 (D8V9E) Rabbit mAb were from Cell Signaling Technology (USA).

### 2.13 Immunofluorescence analysis

For confocal studies, HaCaT cells were grown in a glass chamber and fixed with 10% buffered formalin for 30 minutes, and then incubated with blocking buffer at 4°C overnight. The next day, HaCaT cells were incubated with Anti-Osteopontin antibody (1:500, ab8448, Abcam, USA), GM130 Antibody (1:50, Santa Cruz, USA) for two hours, and incubated with appropriate fluorescence-conjugated secondary antibodies for 1 hour at room temperature. DAPI (Sangon Biotech, China) was used to manifest nuclei. Images were captured using confocal microscopy. Secondary antibodies (Thermo Fisher Scientific, USA) used in immunofluorescence were Alexa Fluor 488 donkey anti-rabbit IgG and Alexa Fluor 555 donkey anti-mouse IgG.

### 2.14 Enzyme-linked immunosorbent assay

The concentrations of OPN in the supernatant of HaCaTs were quantified using the Human Osteopontin/OPN ELISA Kit (KE00233) (Proteintech, USA) according to the protocol provided by the manufacturers.

### 2.15 Statistical analysis

Statistical analysis was performed using GraphPad Prism software (GraphPad Software, USA), and data were presented as the mean ± SEM. All experiments were repeated at least three times unless otherwise mentioned. Data were analyzed for significance using T-test, one-way ANOVA and two-way ANOVA, and the value of P<0.05 was considered to be statistically significant.

## 3 Results

### 3.1 OPN expression is increased in rosacea patients and LL37-induced rosacea inflammation in mice

**Figure 1:**
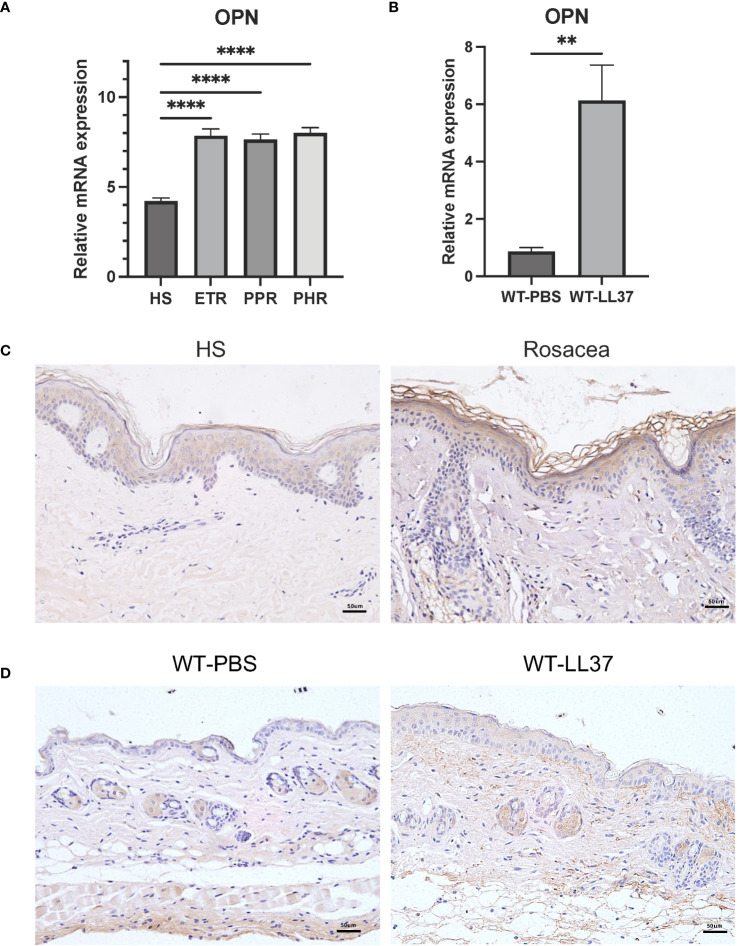
OPN expression in rosacea patients and LL37-induced rosacea-like skin inflammation. **(A)** Relative mRNA expression of OPN was from the GSE65914 dataset, which contains healthy controls (n = 20) and three clinical types of rosacea, including Erythematotelangiectatic (ETR) type (n = 14), papulopustular (PPR) type (n =12), and phytates (PHR) type (n= 12). Data represent the mean ± SEM. One-way ANOVA with Tukey’s *post hoc* test was used for statistical analyses. ****p < 0.0001. **(B)** Relative mRNA expression of OPN in rosacea-like skin lesions of WT mice injected with PBS or LL37 (n = 4 for each group). Data represent the mean ± SEM. T-test was used for statistical analyses. **p < 0.01. **(C)** Representative images of immunohistochemistry of OPN on skin lesions from healthy individuals (HS) and rosacea patients. Scale bar: 50μm. **(D)** Representative images of immunohistochemistry of OPN in skin lesions of WT mice injected with PBS or LL37. Scale bar: 50μm.

To determine the expression pattern of OPN in rosacea, rosacea transcriptome data (GSE65914) was obtained from the GEO database. Firstly, we analyzed the mRNA expression of OPN in rosacea transcriptome data. Results showed that OPN was significantly upregulated in all three types of rosacea (**Figure 1A**). Meanwhile, LL37 was administrated to mice by intradermal injection to induce rosacea-like inflammation (25). Consistently, it was shown that the OPN level was significantly increased in mice injected with LL37 compared with the control group (**Figure 1B**). In addition, IHC results confirmed that OPN protein levels were significantly increased in skin biopsies of rosacea patients (**Figure 1C**) and LL37-induced rosacea-like skin inflammation (**Figure 1D**). Together, these results suggested that OPN has a potential role in rosacea pathogenesis.

### 3.2 OPN promotes LL-37-induced macrophage infiltration and angiogenesis in mice

**Figure 2:**
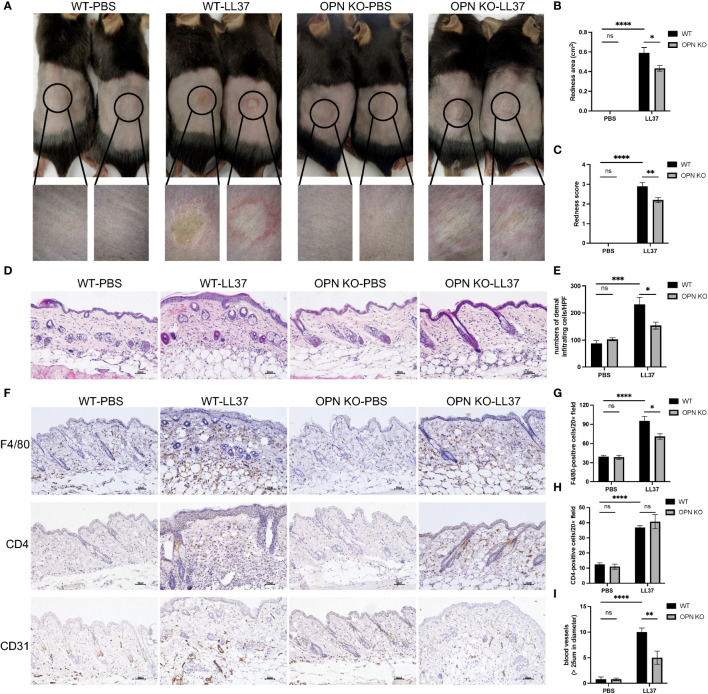
OPN knockdown attenuated rosacea-like skin redness and inflammatory cell infiltration in mice. **(A)** LL37 was injected intradermally into the dorsal skin of WT mice and OPN KO mice to induce a rosacea-like phenotype. The skin lesions were collected two days after injection with PBS or LL37. The lower panel is a magnified image of the circular area in the upper panel. The severity of the rosacea-like phenotypes was assessed according to the redness area **(B)** and redness score **(C)**. Data represent the mean ± SEM. *p < 0.05, **p < 0.01, ****p < 0.0001. Ns, no significance. **(D)** HE staining for the histological analysis of WT mice and OPN KO mice injected with PBS or LL37. Scale bar: 50μm. **(E)** The number of dermal inflammatory infiltration in each group was quantified (n = 4 for each group). Data represent the mean ± SEM. Two-way ANOVA with Tukey’s *post hoc* test was used for statistical analyses. *p < 0.05, ***p < 0.001. Ns, no significance. **(F)** Representative images of immunohistochemistry of F4/80, CD4, and CD31 in WT mice and OPN KO mice injected with PBS or LL37. Scale bar: 50μm. **(G)** The infiltration of F4/80-positive cells was quantified in each group (n = 4 for each group). Data represent the mean ± SEM. Two-way ANOVA with Tukey’s *post hoc* test was used for statistical analyses. *p < 0.05, ****p < 0.0001. Ns, no significance. **(H)** The infiltration of CD4-positive cells was quantified in each group (n = 4 for each group). Data represent the mean ± SEM. Two-way ANOVA with Tukey’s *post hoc* test was used for statistical analyses. ****p < 0.0001. Ns, no significance. **(I)** The number of CD31-positive blood vessels (>25um in diameter) was quantified in each group (n = 4 for each group). Data represent the mean ± SEM. Two-way ANOVA with Tukey’s *post hoc* test was used for statistical analyses. **p < 0.01, ****p < 0.0001. Ns, no significance.

To explore the role of OPN in rosacea, OPN KO mice and WT mice were used to establish LL37-induced rosacea-like skin inflammation models (**Figure 2A**). Rosacea-like features, including the redness area (**Figure 2B**) and redness score (**Figure 2C**), were obviously reduced in OPN KO mice. In addition, histological analysis revealed that the number of inflammatory cell infiltration in the dermis was significantly reduced in OPN KO mice (**Figures 2D, E**). Macrophage infiltration and Th1/Th17 polarized inflammation are considerable symbols in all rosacea subtypes, so we wondered whether OPN regulates cutaneous immune dysfunction in rosacea. As depicted in **Figure 2F**, an obvious accumulation of F4/80^+^ macrophages was observed in LL37-induced rosacea-like skin inflammation, whereas much fewer macrophages were found in OPN KO mice treated with LL37 (**Figure 2G**). Next, we evaluated CD4^+^ T cells in mice skin lesions to determine whether OPN participated in the adaptive immune response. An increasing number of CD4^+^ T cells were observed in LL37-induced skin inflammation. However, there was no significant difference between the OPN KO mice and the WT mice with LL37 treatment (**Figure 2H**). Thus, our data above demonstrated that OPN promotes rosacea-like inflammation through increased infiltration of macrophages and angiogenesis in rosacea.

Angiogenesis and vasodilation were other distinguishing traits of rosacea (26). Therefore, the effect of OPN on angiogenesis in rosacea was assessed by CD31 immunostaining (**Figure 2F**). As illustrated in **Figure 2I**, dermal vessel density was significantly increased in the WT mice, whereas relatively reduced angiogenesis was found in the OPN KO mice. It demonstrated that OPN contributes to rosacea-like inflammation through angiogenesis in rosacea.

### 3.3 OPN promotes the progression of rosacea by regulating the expression of pro-inflammatory factors in the rosacea-like animal model

**Figure 3:**
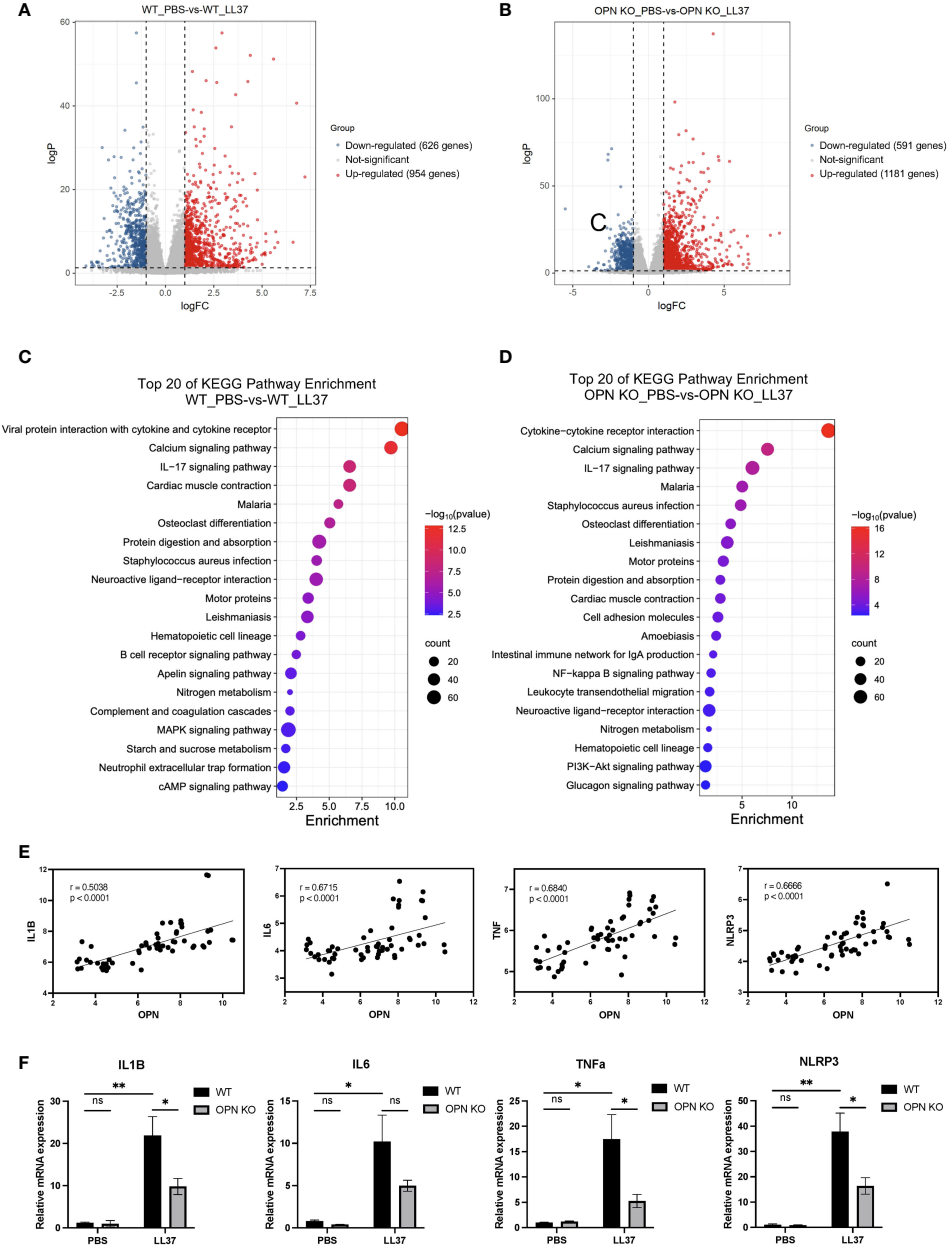
OPN promotes the expression of IL1B in the rosacea-like mice. **(A)** Volcano plots showed the differentially expressed genes between the WT_PBS and WT_LL37 groups. The red, gray, and blue dots represented up-regulated, insignificant, and down-regulated genes, respectively. **(B)** Volcano plots showed the differentially expressed genes between the OPN KO_PBS and OPN KO_LL37 groups. The red, gray, and blue dots represented up-regulated, insignificant, and down-regulated genes, respectively. **(C)** KEGG pathway analysis of DEGs between WT_PBS and WT_LL37 group for functional enrichment analysis. **(D)** KEGG pathway analysis of DEGs between OPN KO_PBS and OPN KO_LL37 groups for functional enrichment analysis. **(E)** Pearson’s correlation analysis was performed to analyze the correlation between the expression level of OPN and IL1B, IL6, TNFA, NLRP3 in GSE65914 rosacea dataset, which includes all the healthy controls (n=20) and rosacea patients (n=38). **(F)** The mRNA expression levels of IL1B, IL6, TNFa, and NLRP3 in skin lesions from WT and OPN KO mice injected with LL37 or PBS (n ≥ 4 for each group). Data represent the mean ± SEM . Two-way ANOVA with Tukey’s *post hoc* test was used for statistical analyses. *p < 0.05, **p < 0.01. Ns, no significance.

To reveal the potential mechanism that OPN promotes rosacea-like inflammation, RNA-seq was performed to identify genes regulated by OPN in rosacea-like skin inflammation. The WT-PBS group and WT-L37 group have 1580 DEGs, including 954 upregulated genes and 626 downregulated genes (|log2FC| ≥ 1, p value < 0.05) (**Figure 3A**). The OPN KO-PBS group and OPN KO-LL37 group have 1772 DEGs, including 1181 upregulated genes and 591 downregulated genes (|log2FC| ≥ 1, p value < 0.05) (**Figure 3B**). The KEGG enrichment analyses have showed the deregulated pathways including IL-17, NF-κB, and MAPK pathways (**Figures 3C, D**), which are known to drive skin inflammation. DEGs from WT group treated or not with LL37 were enriched in MAPK signaling pathway, while DEGs from OPN KO group were not enriched in this signaling pathway. Therefore, more concerned about MAPK signaling pathway had been paid in the following experiments.

IL1B, IL6, NLRP3, and TNFa were important characteristic factors and critical mediators in the inflammation of rosacea (8, 27, 28). Rosacea transcriptome data (GSE65914) was analyzed to further understand the relationship between the expression of OPN and pro-inflammatory factors in healthy controls and rosacea patients. All the samples were included to analyze the correlation between the level of OPN and pro-inflammatory factors. Interestingly, the rosacea patients who expressed higher OPN also expressed higher IL6, IL1B, NLRP3, and TNFa (**Figure 3E**), suggesting that there was a correlation between the expression of OPN and pro-inflammatory factors in rosacea. The pro-inflammatory cytokines including IL1B, NLRP3, and TNFa were also significantly upregulated in rosacea-like skin lesions of WT mice and remarkably downregulated in those of OPN KO mice (**Figure 3F**). Collectively, these results indicated that epidermal OPN contributes to the pathogenesis of rosacea skin disorders by regulating the expression of pro-inflammatory factors.

### 3.4 iOPN promotes the progression of rosacea by regulating IL1B expression via ERK1/2 and JNK signaling pathways in keratinocytes

**Figure 4:**
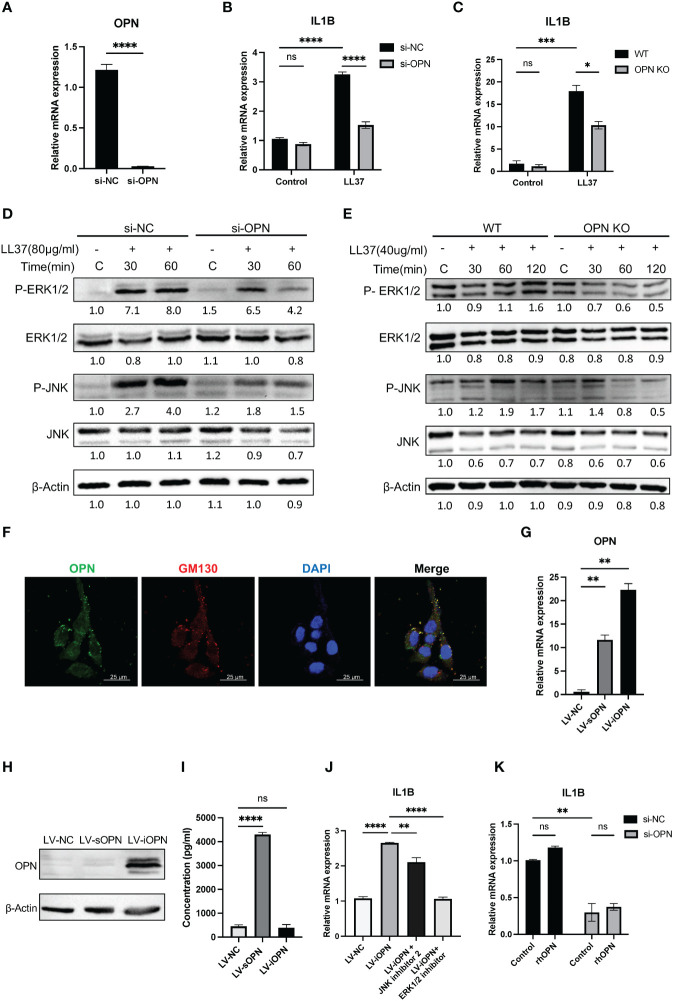
OPN knockdown downregulated the expression of IL1B via ERK1/2 and JNK pathways in keratinocytes. **(A)** The knockdown efficiency of OPN in HaCaT cells was detected by qRT-PCR. Data represent the mean ± SEM. T-test was used for statistical analyses. ****p < 0.0001. **(B)** The mRNA expression level of IL1B in LL37-induced HaCaT cells after si-OPN treatment. Data represent the mean ± SEM. Two-way ANOVA with Tukey’s *post hoc* test was used for statistical analyses. ****p < 0.0001. Ns, no significance. **(C)** The mRNA expression level of IL1B in LL37-induced WT and OPN KO mice primary keratinocytes. Data represent the mean ± SEM. Two-way ANOVA with Tukey’s *post hoc* test was used for statistical analyses. *p < 0.05, ***p < 0.001. Ns, no significance. **(D)** Cultured HaCaT cells treated with si-OPN were stimulated with LL37 (80ug/ml) for various time points. Total cellular proteins were extracted to determine the phosphorylated and total ERK1/2 and JNK by western blot. β-Actin was selected as an internal control. **(E)** WT and OPN KO mice primary keratinocytes were stimulated with LL37 (40 ug/ml) for various time points. Total cellular proteins were extracted to determine the phosphorylated and total ERK1/2 and JNK by western blot. β-Actin was selected as an internal control. **(F)** Co-localization of OPN and GM130 in HaCaT cells was observed by confocal microscopy. Green indicates OPN. Red indicates GM130. Blue indicates DAPI. Scale bar, 25 μm. **(G)** The overexpression efficiency of sOPN and iOPN in HaCaT cells was detected by qRT-PCR. Data represent the mean ± SEM. One-way ANOVA with Dunnett’s *post hoc* test was used for statistical analyses. **p < 0.0001. **(H)** The overexpression levels of OPN in HaCaT cells were detected by Western blotting. β-Actin was selected as an internal control. **(I)** The overexpression levels of sOPN in the supernatant of HaCaT cells were detected by ELISA. Data represent the mean ± SEM. One-way ANOVA with Tukey’s *post hoc* test was used for statistical analyses. ****p < 0.0001. Ns, no significance. **(J)** LV-iOPN HaCaTs were treated with ERK1/2 inhibitor (SCH772984) (200nM) and JNK inhibitor II (100nM) for 20 hours, and the mRNA expression level of IL1B was detected by qRT-PCR. Data represent the mean ± SEM. One-way ANOVA with Tukey’s *post hoc* test was used for statistical analyses. **p <0.01, ****p < 0.0001. **(K)** HaCaT cells with si-OPN treatment were stimulated with rhOPN (2500ng/ml) in serum-free medium, and the mRNA expression level of IL1B was detected by qRT-PCR. Data represent the mean ± SEM. Two-way ANOVA with Tukey’s *post hoc* test was used for statistical analyses. **p <0.01. Ns, no significance.

Keratinocytes are the most prominent cells within the epidermis. To further understand how OPN functions in keratinocytes, a specific siRNA targeting OPN gene was transfected into keratinocytes. The knockdown efficiency of si-OPN was up to 90% in the HaCaT cells (P < 0.0001) (**Figure 4A**). Consistent with the result *in vivo*, OPN deletion markedly inhibited the expression of LL37-induced pro-inflammatory cytokine IL1B in both HaCaTs (**Figure 4B**) and primary mouse keratinocytes (**Figure 4C**). As shown in **Figures 4D, E**, LL37 treatment significantly increased the phosphorylated ERK1/2 and JNK in both HaCaTs and primary mouse keratinocytes, whereas OPN knockdown significantly decreased p-ERK/ERK and p-JNK/JNK ratios (**Figures S2A, B**).

To analyze the expression pattern of OPN in keratinocytes, dual immunofluorescence staining of OPN and Golgi matrix protein 130 (GM130) was performed. It was shown that HaCaT contains both secretory and intracellular forms of OPN (**Figure 4F**). Next, we sought to determine whether sOPN or iOPN regulates inflammatory signaling pathways. The lentivirus-mediated overexpression of sOPN and iOPN was constructed in HaCaT cells, and the expression level of OPN mRNA was detected in these infected cells (**Figure 4G**). The intracellular and extracellular OPN overexpression levels were detected by western blot and ELISA respectively. Results showed that the intracellular protein level was remarkably increased in LV-iOPN HaCaTs (**Figure 4H**), and OPN concentration in the supernatant was significantly increased in the LV-sOPN HaCaTs (**Figure 4I**). It was confirmed that overexpression of iOPN significantly increased the expression of pro-inflammatory IL1B in HaCaT cells. Meanwhile, both the ERK1/2 inhibitor (SCH772984) and JNK inhibitor II effectively inhibited the expression of *IL1B* in LV-iOPN HaCaTs (**Figure 4J**).

Because LV-sOPN HaCaTs show slight expression of intracellular OPN (**Figure 4H**), it was not appropriate for further identification of the role of sOPN in the IL1B expression. Instead, rhOPN was added to si-OPN-treated HaCaTs, of which the intracellular OPN was almost eliminated. It was found that extracellular OPN could not increase the expression of IL1B (**Figure 4K**). Together, these findings demonstrated that iOPN promotes the progression of rosacea by regulating IL1B expression via ERK1/2 and JNK signaling pathways in keratinocytes.

### 3.5 OPN knockdown inhibits the polarization of M1 macrophages in rosacea lesions

**Figure 5:**
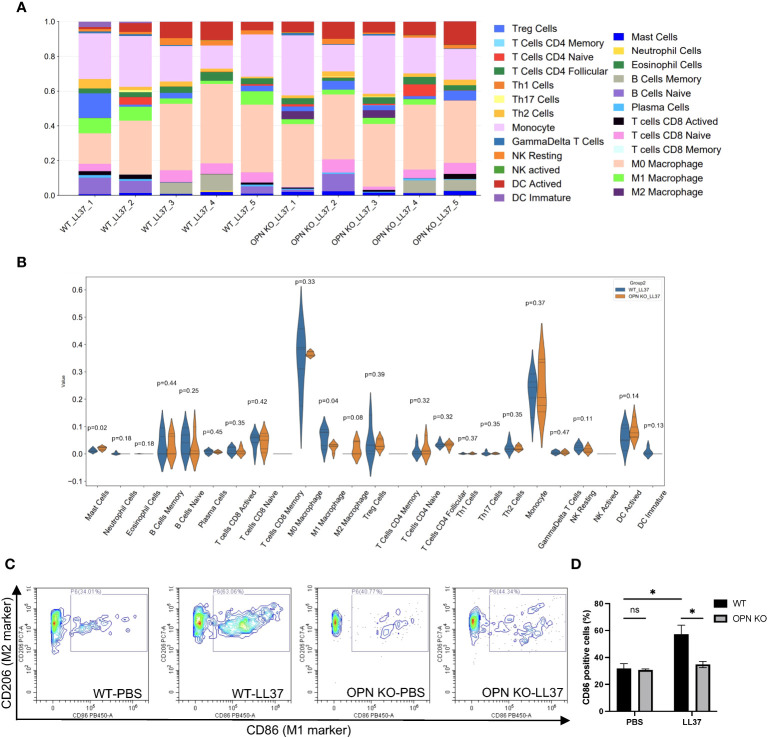
OPN knockdown inhibited M1 macrophage polarization in LL37-induced rosacea mice. **(A)** Analysis of immune cells infiltration using CIBERSORT in skin lesions from WT mice and OPN KO mice injected with LL37 (n = 5 for each group). **(B)** Statistical analysis of immune cells infiltration in WT-LL37 group (blue bar) and OPN KO-LL37 group (yellow bar) was shown in the violin plot. T-test was used for statistical analyses. **(C)** Flow cytometry was performed to quantitatively analyze the percentage of CD86-positive cells in skin lesions of the WT-PBS group (n = 3), OPN KO-PBS (n = 3), WT-LL37 group (n = 6), and OPN KO-LL37 group (n = 6). CD86 was identified as an M1 macrophage marker and CD206 was identified as an M2 macrophage marker. **(D)** CD86-positive cells in each group were quantified. Data represent the mean ± SEM. Two-way ANOVA followed by Sidak’s multiple comparison test was used for statistical analyses. *p < 0.05. Ns, no significance.

The polarization of M1 macrophages promoted rosacea inflammation. M1 macrophage infiltration was significantly increased in rosacea and positively correlated with CEA and IGA scores (15, 29). Our results revealed that OPN promoted the infiltration of F4/80+ macrophages in LL37-induced rosacea-like skin inflammation. To further investigate the role of OPN in immune cell infiltration, CIBERSORT was used to assess immune cell infiltration in lesional tissues from rosacea-like mice based on our transcriptome data (**Figure 5A**). Compared with the WT_LL37 group, the percentage of M1 macrophage infiltration in OPN KO_LL37 group was significantly reduced (**Figure 5B**). Next, flow cytometry were performed to quantitatively analyze the percentage of M1 and M2 macrophages in LL37-induced rosacea-like lesions from WT and OPN KO mice, with gating strategy provided in **Figure S3A**. Phenotypically, M1 macrophage expressed higher levels of CD86, whereas CD206 was commonly associated with M2 macrophage (30). Flow cytometry analysis showed that compared with the PBS group, the percentage of CD86-positive M1 macrophages increased from 32% to 57% of total macrophages in rosacea-like lesions of WT mice, whereas the percentage of the CD86+ macrophages increased from 31% to 35% in OPN KO mice (**Figures 5C, D**). However, there was no significant difference in the percentage of CD206-positive cells (**Figures S3B, C**). Together, these results indicated that OPN promotes the M1 polarization of macrophages in the rosacea-like mice model.

### 3.6 Keratinocytes-derived sOPN promotes M1 macrophage polarization

**Figure 6:**
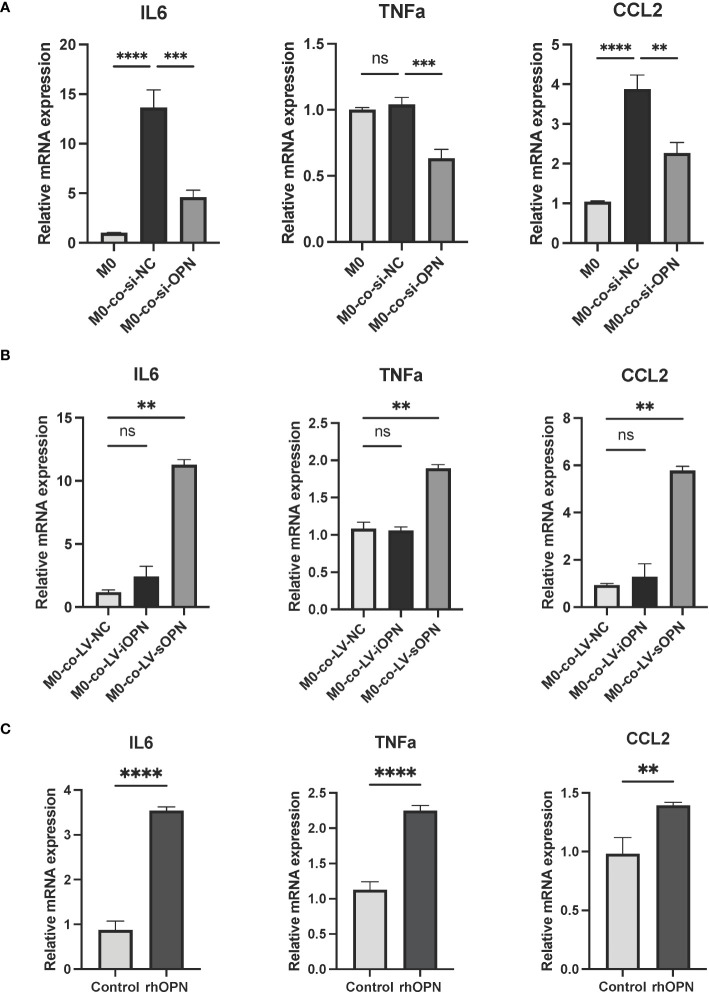
OPN secreted by LL37-induced keratinocytes promoted M1 macrophage polarization. **(A)** THP-1-derived macrophages (M0) were co-cultured with LL37-induced and si-OPN-treated HaCaTs for 48 hours, and the mRNA expression levels of IL6, TNFa, and CCL2 were determined by qRT-PCR. Data represent the mean ± SEM. One-way ANOVA with Tukey’s *post hoc* test was used for statistical analyses. **p < 0.01, ***p < 0.001, ****p < 0.0001. Ns, no significance. **(B)** THP-1-derived macrophages (M0) were co-cultured with LL37-induced LV-NC HaCaTs, LV-sOPN HaCaTs, and LV-iOPN HaCaTs for 48 hours, and the mRNA expression levels of IL6, TNFa, and CCL2 were determined by qRT-PCR. Data represent the mean ± SEM. One-way ANOVA with Tukey’s *post hoc* test was used for statistical analyses. **p < 0.01. Ns, no significance. **(C)** THP-1-derived macrophages(M0) were stimulated with rhOPN for 48 hours, and the mRNA expression levels of IL6, TNFa, and CCL2 were determined by qRT-PCR. Data represent the mean ± SEM. T-test was used for statistical analyses. **p < 0.01, ****p < 0.0001.

Keratinocyte-immune cell crosstalk has been considered to contribute to skin homeostasis (31). To explore whether the activated keratinocytes affect the differentiation of the macrophages in rosacea, THP-1-derived macrophages (M0) were co-cultured with LL37-induced HaCaTs for 48 hours. Co-cultured with si-OPN-treated HaCaTs significantly inhibited the mRNA expression levels of the M1 macrophage markers (**Figure 6A**). Since OPN deficiency eliminates both intracellular and secreted forms of OPN, the regulation role of OPN in macrophage polarization still needs to be investigated in depth. It was wondered whether the extracellular OPN affects the polarization of the macrophages. The results showed that co-cultured with LV-sOPN HaCaTs, not LV-iOPN HaCaTs, significantly increased the mRNA expression levels of the M1 macrophage markers, including IL6, CCL2, and TNFa (**Figure 6B**). Moreover, as extracellular OPN *in vitro*, rhOPN was added to macrophages (M0). It was found that rhOPN potentiated the expression of M1 macrophage signature genes (**Figure 6C**). These findings indicated that sOPN plays a vital role in keratinocyte-macrophage crosstalk. Communication between keratinocytes and immune cells may provide new insight into the pathogenesis of rosacea.

## 4 Discussion

Rosacea is a chronic inflammatory facial disease occurring worldwide. It was well recognized that dysregulation of the innate and adaptive immune response as well as neurovascular dysregulation contribute to rosacea pathophysiology significantly (32). The expression of cytokines including IL-1β, IL-6, and IL-17, was associated with PPR type (5, 8, 33). Multiple studies suggested that IL-1β may be a major driver of rosacea for its increased expression (8). In this study, OPN was an inducer for the production of pro-inflammatory cytokine IL-1β in rosacea pathogenesis. Besides, it was reported that IL-1β also interacts with pro-angiogenic factor VEGF in angiogenic responses. Overall, increased expression of IL-1β may contribute to the aggravation of rosacea inflammation and blood vessel formation.

OPN plays a vital role in increasing inflammatory cell infiltration and promoting inflammation in acute and chronic inflammatory diseases (34). Although OPN was generally classified as a pro-inflammatory cytokine, it may also had anti-inflammatory effects. OPN inhibited the expression of iNOS by promoting the degradation of Stat1 in macrophages (35, 36). Most studies regarded OPN as a secreted protein, that binds to integrins or CD44 (37) receptors and activates multiple signaling pathways, including NFκB and MAPK signaling pathways (38, 39). However, an alternative translation of different start codons from a single OPN mRNA can generate another OPN isoform called iOPN (40). These OPN isoforms may exist in different cells and mediate different biological functions. Natural killer (NK) cells expressed higher levels of iOPN, and iOPN deficiency promoted NK cell death and impaired NK cell differentiation (41). In anti-fungal responses, iOPN was involved in the TLR2 pathway and enhanced MAPK activation (42). Our study reveals that keratinocyte contains both sOPN and iOPN. Instead of sOPN, iOPN upregulates the expression of cytokine IL-1β via ERK1/2 and JNK signaling pathways in LL37-induced inflammation. iOPN plays a pro-inflammatory role and contributes to rosacea pathogenesis.

Activation and infiltration of macrophages have been reported in the skin lesions of rosacea patients (5). It was found that M1 macrophage polarization promoted rosacea inflammation (15, 29). M1 macrophage polarization in rosacea may be the result of multiple factors. Previous studies showed that GBP5 and ADAMDEC1 played a pro-inflammatory role in rosacea-like skin inflammation by regulating the M1 macrophage polarization (15, 29). However, OPN plays an increasingly important role in macrophage polarization, and whether or not OPN regulates macrophage polarization remains controversial (43). Recently, it was reported that macrophage polarity defined by CXCL9 and OPN expression, rather than traditional M1 and M2 markers, was strongly associated with tumor prognosis (44). In nonalcoholic fatty liver disease, OPN promoted macrophage M1 polarization by activation of the JAK1/STAT1/HMGB1 signaling pathway (45). Another study indicated that OPN served a protective role and downregulated M1 macrophage markers in hypertension and vascular calcification (46). In the present study, we demonstrated that OPN promoted macrophage infiltration and M1 macrophage polarization in rosacea lesions, which may be one of the important factors contributing to the exacerbation of rosacea inflammation.

Since OPN deficiency in keratinocytes eliminates both intracellular and secreted forms of OPN, the regulation role of sOPN and iOPN in macrophage polarization still needs to be investigated in depth. Previous studies have highlighted that dynamic epithelial-immune crosstalk fine-tunes epithelial homeostasis (47). In rosacea skins, infiltrated immune cells produce inflammatory mediators that lead to the activation of keratinocytes (48). The activated keratinocytes may further affect the polarization of the macrophages. In this study, the co-culture assay was performed to investigate whether sOPN or iOPN affected keratinocyte-macrophage crosstalk in rosacea inflammation. It was identified that instead of iOPN, keratinocyte-derived sOPN played a crucial role in promoting M1 macrophage polarization *in vitro*. It was shown that iOPN promoted IL1B expression in keratinocytes. IL1B was one of the M1 macrophage marker. However, the role of extracellular IL1B in macrophage polarization remained unclear. RhOPN was added to si-OPN-treated HaCaTs to study the role of sOPN in keratinocytes. The results showed that sOPN could not promote IL1B expression in keratinocytes. Similarly, the level of IL1B expression was not changed in macrophages treated with rhOPN. Moreover, some inflammatory mediators regulated by iOPN may also indirectly affect macrophage polarization and need to be further clarified. Some studies have revealed IL-1, IL-10, IL-12, IL-17, and IFN-γ (37, 49), of which secretion was regulated by OPN in different cells and tissues. Disruption of the crosstalk between keratinocytes and immune cells is expected to be a promising therapeutic target for rosacea treatment. In addition, sOPN or iOPN-specific knock-in mice will be helpful to better understand the different roles of iOPN and sOPN *in vivo*.

**Figure 7:**
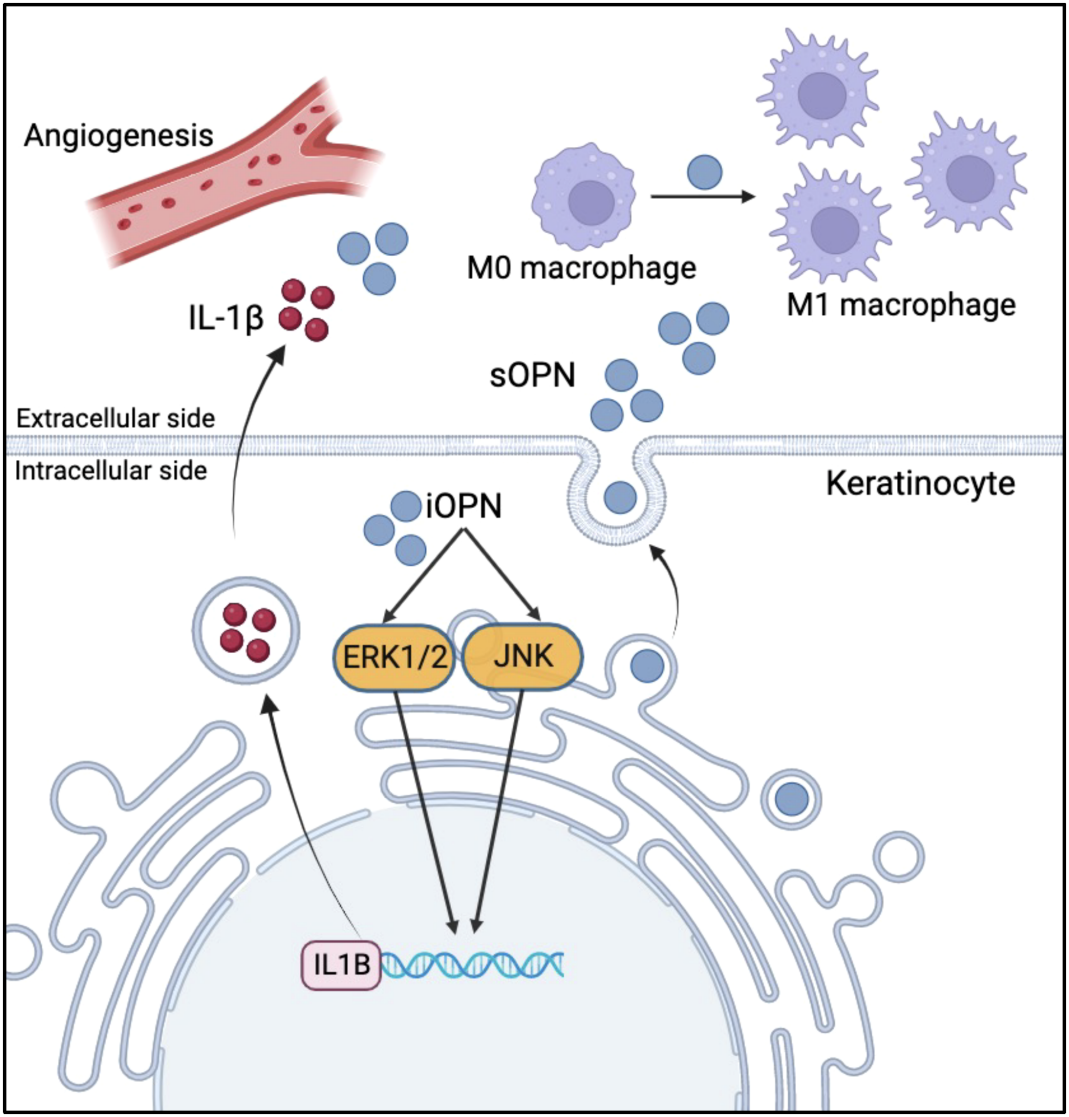
Schematic diagram of sOPN and iOPN in the pathogenesis of rosacea. sOPN and iOPN play different roles in the pathogenesis of rosacea. iOPN plays a crucial role in the upregulation of pro-inflammatory cytokine IL1B expression via ERK1/2 and JNK signaling pathways. sOPN participates in keratinocyte-macrophage crosstalk by promoting M1 macrophage polarization.

In conclusion, our study sheds light on the pivotal role of sOPN and iOPN in the pathogenesis of rosacea (**Figure 7**). Understanding the role of iOPN and sOPN may provide evidence for their use as biomarkers and therapeutic targets in rosacea.

## Data availability statement

The datasets of GSE65914 can be found in online repositories. Mice RNA-seq gene expression data can be found in the **Supplementary Material**.

## Ethics statement

The studies involving humans were approved by Medical Ethics Committee of Peking University Shenzhen Hospital. The studies were conducted in accordance with the local legislation and institutional requirements. The participants provided their written informed consent to participate in this study. The animal study was approved by Shenzhen Perking University - the Hong Kong University of Science and Technology Medical Center Animal Care and Use Committee. The study was conducted in accordance with the local legislation and institutional requirements. Written informed consent was obtained from the individual(s) for the publication of any potentially identifiable images or data included in this article.

## Author contributions

ST: Data curation, Formal analysis, Methodology, Writing – original draft. HH: Data curation, Methodology, Writing – original draft. ML: Methodology, Writing – review & editing. KZ: Data curation, Methodology, Writing – original draft. QW: Funding acquisition, Methodology, Writing – review & editing. XL: Data curation, Methodology, Writing – original draft. LW: Conceptualization, Methodology, Writing – review & editing. BY: Funding acquisition, Supervision, Writing – review & editing. XC: Conceptualization, Funding acquisition, Methodology, Writing – review & editing.
