# Corrigendum: OPN promotes pro-inflammatory cytokine expression via ERK/JNK pathway and M1 macrophage polarization in Rosacea

**DOI:** 10.3389/fimmu.2024.1382092

**Published:** 2024-02-29

**Authors:** Siyi Tang, Hao Hu, Manhui Li, Kaoyuan Zhang, Qi Wu, Xiaojuan Liu, Lin Wu, Bo Yu, Xiaofan Chen

**Affiliations:** ^1^ Shenzhen Key Laboratory for Translational Medicine of Dermatology, Biomedical Research Institute, Shenzhen Peking University - The Hong Kong University of Science and Technology Medical Center, Shenzhen, Guangdong, China; ^2^ Department of Dermatology, Peking University Shenzhen Hospital, Shenzhen, China; ^3^ Greater Bay Biomedical Innocenter, Shenzhen Bay Laboratory, Shenzhen, China

**Keywords:** Rosacea, OPN, keratinocyte, macrophage, inflammation

In the published article, there was an error in **Figure 2A** as published. The magnified image for the WT-PBS group was misused in the OPN KO-PBS group in the lower panel of **Figure 2A**. The corrected **Figure 2** and its caption appear below.

**Figure 2:**
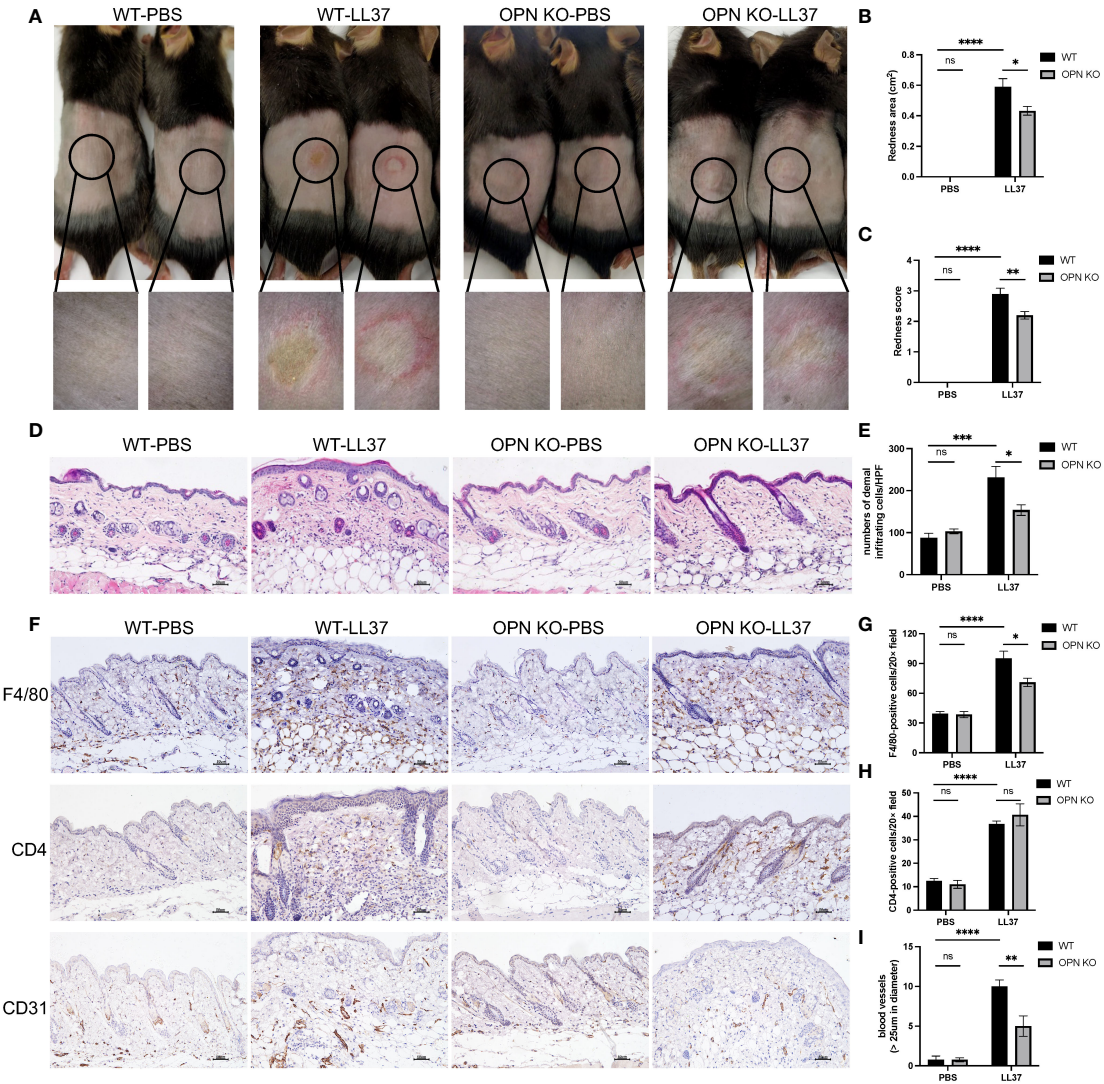
OPN knockdown attenuated rosacea-like skin redness and inflammatory cell infiltration in mice. **(A)** LL37 was injected intradermally into the dorsal skin of WT mice and OPN KO mice to induce a rosacea-like phenotype. The skin lesions were collected two days after injection with PBS or LL37. The lower panel is a magnified image of the circular area in the upper panel. The severity of the rosacea-like phenotypes was assessed according to the redness area **(B)** and redness score **(C)**. Data represent the mean ± SEM. *p < 0.05, **p < 0.01, ****p < 0.0001. Ns, no significance. **(D)** HE staining for the histological analysis of WT mice and OPN KO mice injected with PBS or LL37. Scale bar: 50μm. **(E)** The number of dermal inflammatory infiltration in each group was quantified (n = 4 for each group). Data represent the mean ± SEM. Two-way ANOVA with Tukey’s *post hoc* test was used for statistical analyses. *p < 0.05, ***p < 0.001. Ns, no significance. **(F)** Representative images of immunohistochemistry of F4/80, CD4, and CD31 in WT mice and OPN KO mice injected with PBS or LL37. Scale bar: 50μm. **(G)** The infiltration of F4/80-positive cells was quantified in each group (n = 4 for each group). Data represent the mean ± SEM. Two-way ANOVA with Tukey’s *post hoc* test was used for statistical analyses. *p < 0.05, ****p < 0.0001. Ns, no significance. **(H)** The infiltration of CD4-positive cells was quantified in each group (n = 4 for each group). Data represent the mean ± SEM. Two-way ANOVA with Tukey’s *post hoc* test was used for statistical analyses. ****p < 0.0001. Ns, no significance. **(I)** The number of CD31-positive blood vessels (>25um in diameter) was quantified in each group (n = 4 for each group). Data represent the mean ± SEM. Two-way ANOVA with Tukey’s *post hoc* test was used for statistical analyses. **p < 0.01, ****p < 0.0001. Ns, no significance.

The authors apologize for this error and state that this does not change the scientific conclusions of the article in any way. The original article has been updated.

