# Supplementary material for: OPN promotes pro-inflammatory cytokine expression via ERK/JNK pathway and M1 macrophage polarization in Rosacea
Source: Front Immunol. 2024 Jan 5;14:1285951. doi: 10.3389/fimmu.2023.1285951 (PMC10796667; doi:10.3389/fimmu.2023.1285951)
Supplement: Supplementary file 2 [file DataSheet_2.docx]

Supplementary Material

# Supplementary Table1

| **Target gene** | **Forward primers Reverse primers** |
| --- | --- |
| Human OPN-F | GCTGATTCTGGAAGTTCTGAGGA |
| Human OPN-R | GGACTTACTTGGAAGGGTCTCT |
| Human sOPN-F | atttccggtgaattcctcgagATGAGAATTGCAGTGATTTGCTTT |
| Human sOPN-R | ggagggagaggggcgggatccTTAATTGACCTCAGAAGATGCACTATC |
| Human iOPN-F | atttccggtgaattcctcgagATGATACCAGTTAAACAGGCTGATTCTGG |
| Human iOPN-R | ggagggagaggggcgggatccTTAATTGACCTCAGAAGATGCACTATC |
| Human IL6-F | ACTCACCTCTTCAGAACGAATTG |
| Human IL6-R | CCATCTTTGGAAGGTTCAGGTTG |
| Human Actin-F | CACCATTGGCAATGAGCGGTTC |
| Human Actin-R | AGGTCTTTGCGGATGTCCACGT |
| Human CCL2-F | AGAATCACCAGCAGCAAGTGTCC |
| Human CCL2-R | TCCTGAACCCACTTCTGCTTGG |
| Human IL1B-F | ATGATGGCTTATTACAGTGGCAA |
| Human IL1B-R | GTCGGAGATTCGTAGCTGGA |
| Human TNFa-F | CTCTTCTGCCTGCTGCACTTTG |
| Human TNFa-R | ATGGGCTACAGGCTTGTCACTC |
| Mouse IL6-F | TACCACTTCACAAGTCGGAGGC |
| Mouse IL6-R | CTGCAAGTGCATCATCGTTGTTC |
| Mouse IL1B-F | TGGACCTTCCAGGATGAGGACA |
| Mouse IL1B-R | GTTCATCTCGGAGCCTGTAGTG |
| Mouse TNFa-F | GGTGCCTATGTCTCAGCCTCTT |
| Mouse TNFa-R | GCCATAGAACTGATGAGAGGGAG |
| Mouse NLRP3-F | TCACAACTCGCCCAAGGAGGAA |
| Mouse NLRP3-R | AAGAGACCACGGCAGAAGCTAG |
| Mouse Actin-F | CATTGCTGACAGGATGCAGAAGG |
| Mouse Actin-R | TGCTGGAAGGTGGACAGTGAGG |
| Mouse OPN-F | GCTTGGCTTATGGACTGAGGTC |
| Mouse OPN-R | CCTTAGACTCACCGCTCTTCATG |

# Supplementary Figure

#
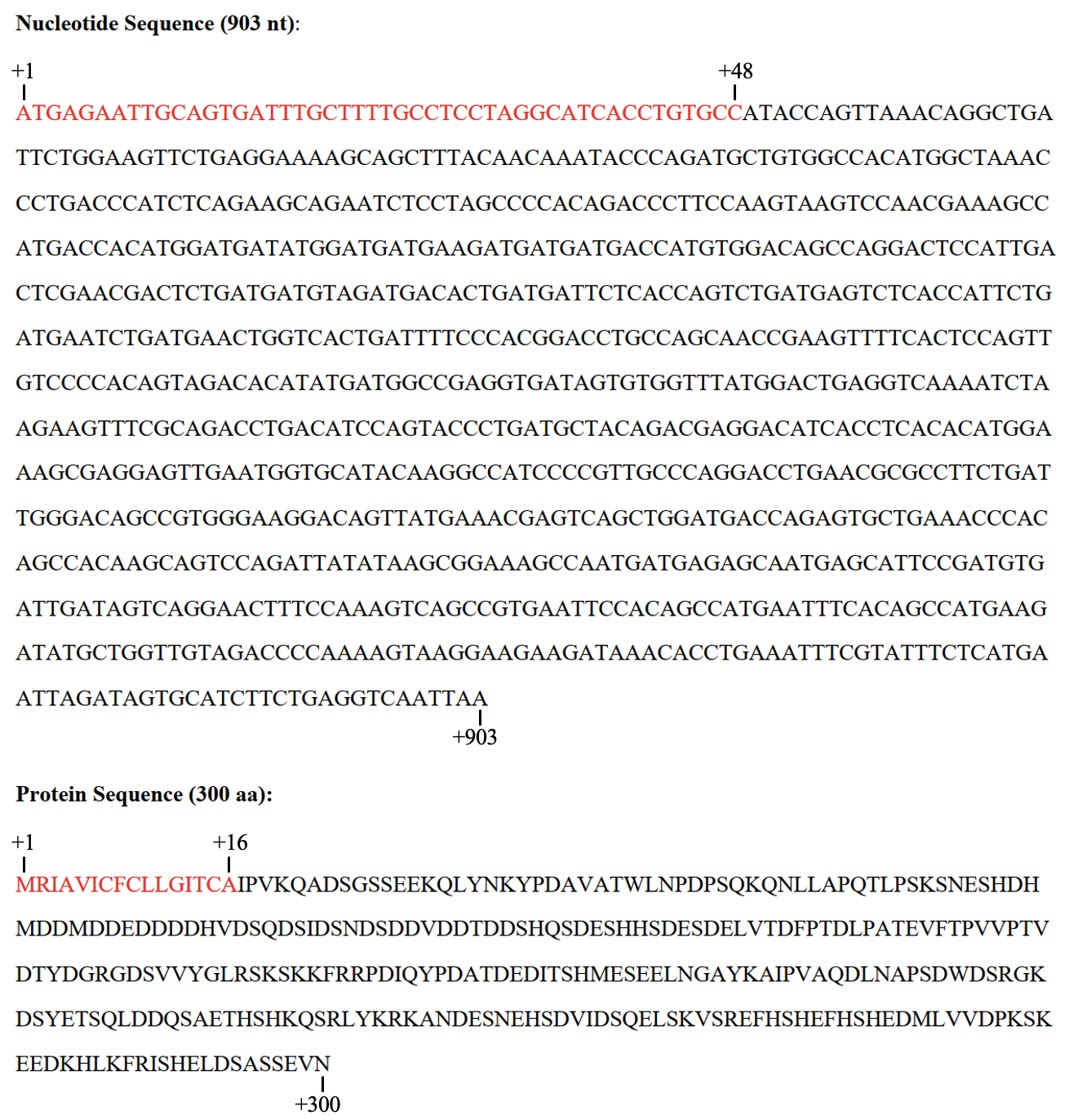


**Supplementary Figure 1**. The sOPN nucleotide sequence (903 nt) was translated into the OPN peptide, which encoded 300 amino acids (aa) and a termination codon. The signal peptide is a short peptide located in the N-terminal of this protein that carries information for protein secretion. Red letters represent the 16 aa signal peptide and its 48 nucleotide sequence. iOPN plasmid was constructed that lacked the signal peptide of sOPN but had an artificial ATG translation start codon inserted.


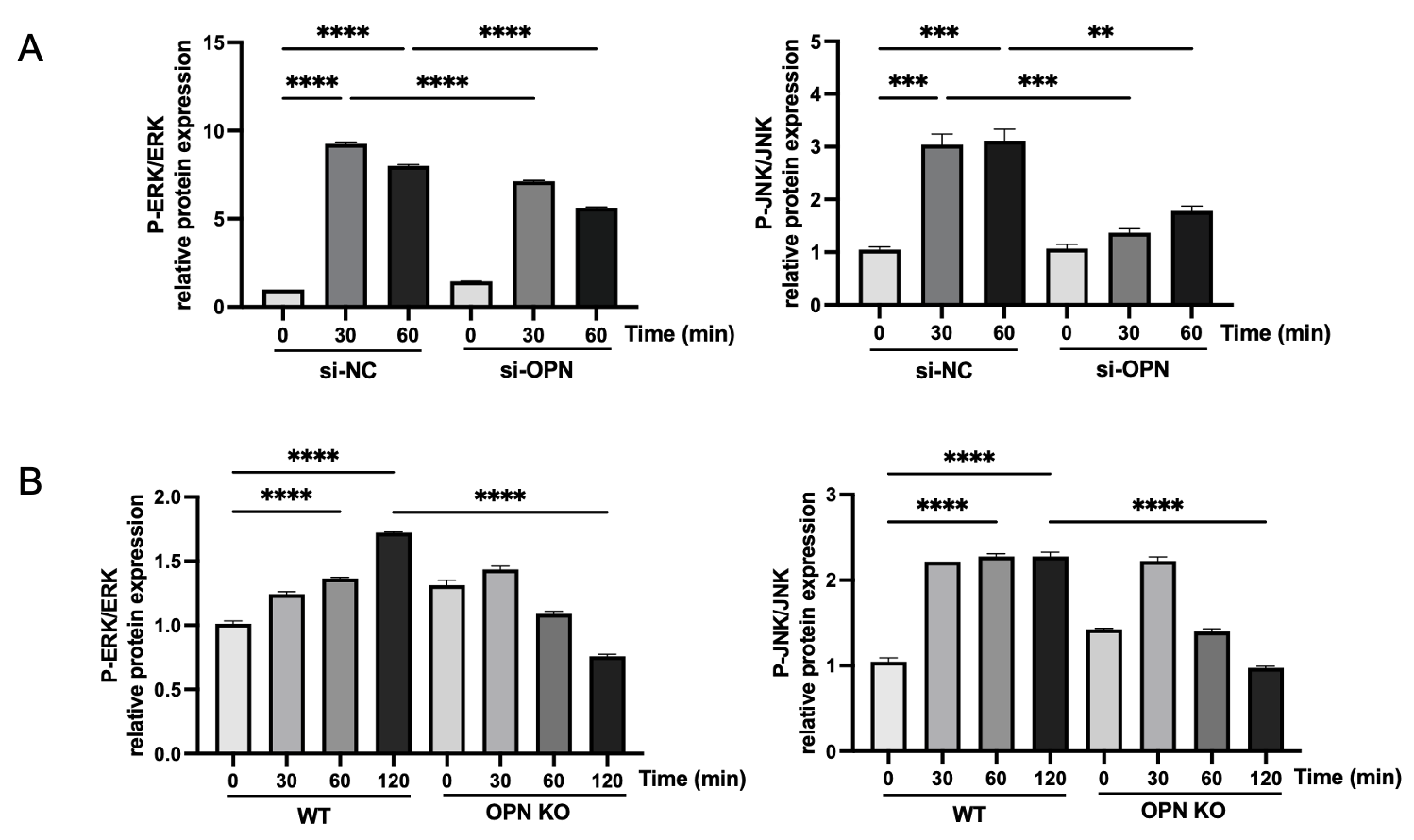


**Supplementary Figure 2.** **﻿** (A) The quantification and statistical analysis for P-ERK/ERK and P-JNK/JNK levels in Figure 4D. ﻿One-way ANOVA followed by Tukey's multiple comparison test was used for statistical analyses. ****p < 0.0001, ***p < 0.001, **p < 0.01. (B) The quantification and statistical analysis for P-ERK/ERK and P-JNK/JNK levels in Figure 4E. ﻿ One-way ANOVA followed by Tukey's multiple comparison test was used for statistical analyses. ****p < 0.0001.

**
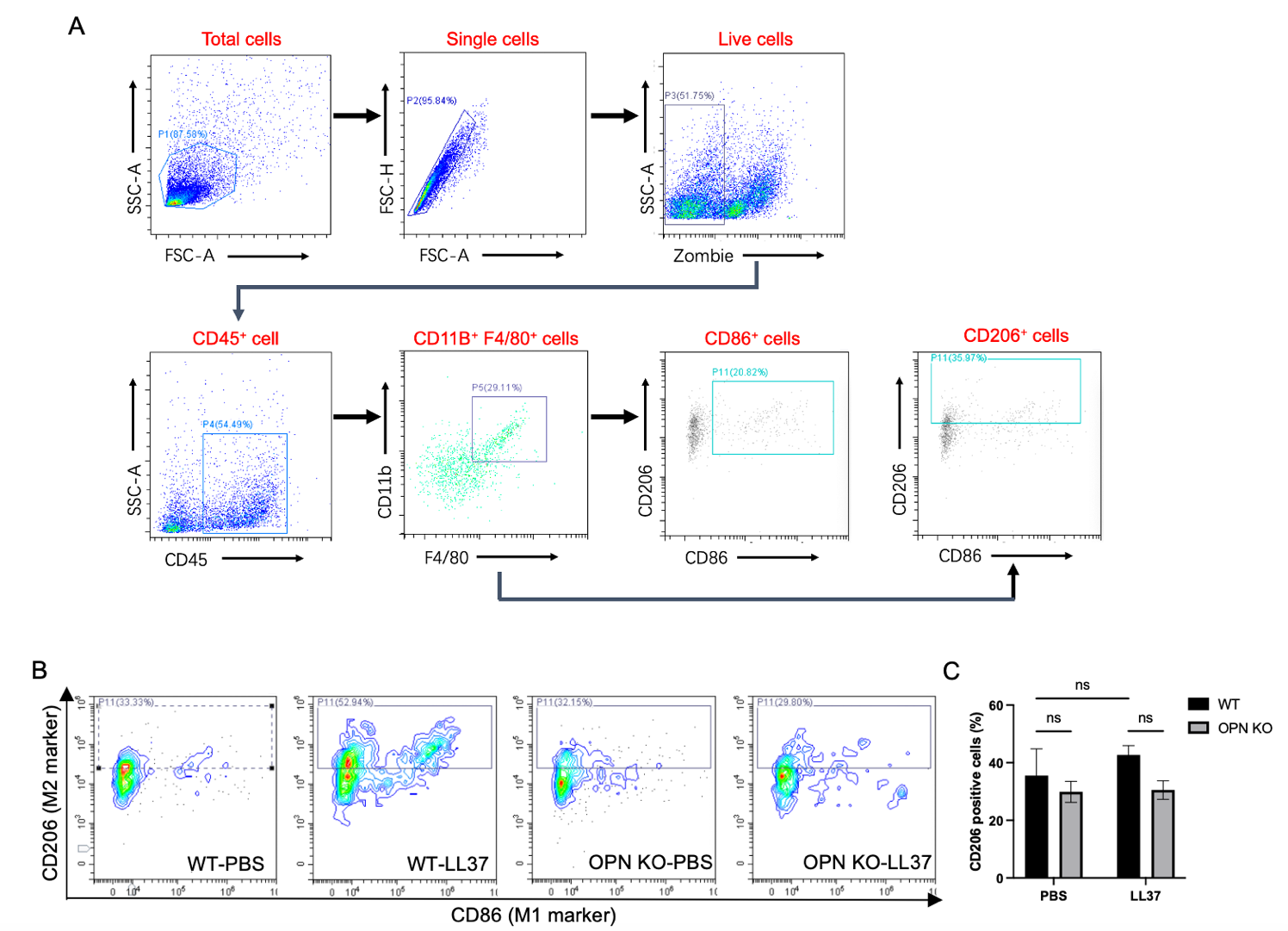
**

**Supplementary Figure 3.** (A) The flow cytometry of macrophage gating strategy in LL37-induced rosacea lesions. (B) Flow cytometry was performed to quantitatively analyze the CD206-positive cells in skin lesions of the WT-PBS group (n = 3), OPN KO-PBS (n = 3), WT-LL37 group (n = 6), OPN KO-LL37 group (n = 6). CD86 was identified as an M1 macrophage marker and CD206 was identified as an M2 macrophage marker. (C) The CD206-positive cells in each group were quantified. Data represent the mean ± SEM. ﻿Two-way ANOVA followed by Tukey's multiple comparison test was used for statistical analyses. Ns, no significance.
